# Recurrence of paratesticular liposarcoma: a case report and review of the literature

**DOI:** 10.1186/1477-7819-12-276

**Published:** 2014-08-29

**Authors:** Raimondo Gabriele, Giuseppe Ferrara, Maria Rita Tarallo, Alessio Giordano, Antonietta De Gori, Luciano Izzo, Marco Conte

**Affiliations:** Department of Surgery, ‘P.Valdoni’ – University of Rome ‘LaSapienza’, Rome, Italy

**Keywords:** Paratesticular liposarcoma, Spermatic cord sarcoma, Inguinal canal tumors

## Abstract

Paratesticular liposarcomas are rare tumors that typically affect adult. Diagnosis is very difficult and inadequate surgical excision leads to a high rate of recurrence.

We report a case of local recurrence of paratesticular liposarcoma diagnosed six months following surgery.

Since there is low response to adjuvant treatments, extensive surgery remains the only curative approach, as shown by the case described here and the following review of the literature.

## Background

Paratesticular liposarcomas (PLs), first reported in 1952, are rare tumors that comprise approximately 3 to 7% of all paratesticular sarcomas [1, 2], and typically affect adults aged 50 to– 60 years [3]. These tumors may arise from the adipose tissue around the spermatic cord or by malignant transformation of a pre-existing lipoma [4, 5]. Preoperative diagnosis is uncertain due to the rarity of these malignancies. Such tumors are often treated by inadequate surgical excision with a subsequent high rate of recurrence.

In this report, the authors report a case of local recurrence of PL diagnosed six months following inadequate surgical excision.

## Case presentation

**Figure 1:**
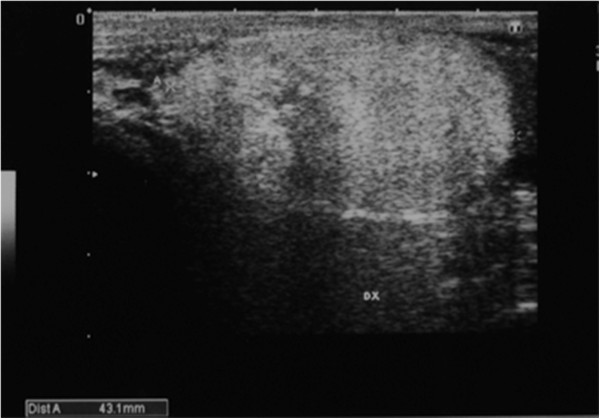
**Ultrasound scan showing a 4.3 × 2.3 cm non** **-homogeneous right scrotal mass.**

A 50-year-old man, who had undergone right spermacelectomy in 2003, was admitted, in March 2011, to the General Surgery Department of the University of Roma ‘Sapienza’. He presented with a slowly growing mass located in his right hemiscrotum, with no other signs or symptoms. A physical examination demonstrated a right intrascrotal swelling, about 5 cm in maximum diameter, soft in consistency, with no pain and negative transillumination; the inguinal lymph nodes were nonpalpable. There were no specific abnormalities in the laboratory findings. An ultrasound (US) scan showed a 4.3 × 2.3 cm nonhomogeneous right scrotal lesion, diagnosed as a lipoma (Figure 1).

Surgical exploration revealed a 5 × 3 cm solid mass arising from his right epididymus. The mass did not involve the didymus, the spermatic cord or surrounding tissues. A wide local excision was performed. A frozen section showed free surgical margins (R0 resection).

The surgical specimen consisted of an irregular, seemingly circumscribed mass of yellow fat intermingled with a small amount of greyish, gelatinous zones.

Microscopically, predominantly mature, adult-type fat cells were shown. Scattered among them were spindled atypical cells with enlarged, hyperchromatic, irregular-shaped nuclei and multivacuolated lipoblasts.

Neither inflammatory cells nor dense fibrotic zones were found and a diagnosis of well- differentiated ‘lipoma-like’ liposarcoma was made.

**Figure 2:**
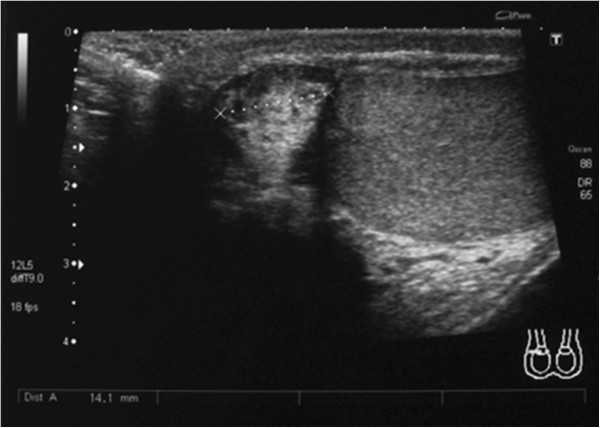
**Ultrasound scan of the right hemiscrotum showing a nonhomogeneous 1.5 × 2 cm mass with no evidence of tissue infiltration or pathological lymph nodes.**

A monthly follow-up was carried out. Six months later, at the US scan of the right hemiscrotum, a nonhomogeneous 1.5 × 2 cm mass was revealed, with no evidence of tissue infiltration or pathological lymph nodes (Figure 2). A second surgical procedure of radical orchiectomy and funicolectomy, via the right inguinal approach, was performed.

**Figure 3:**
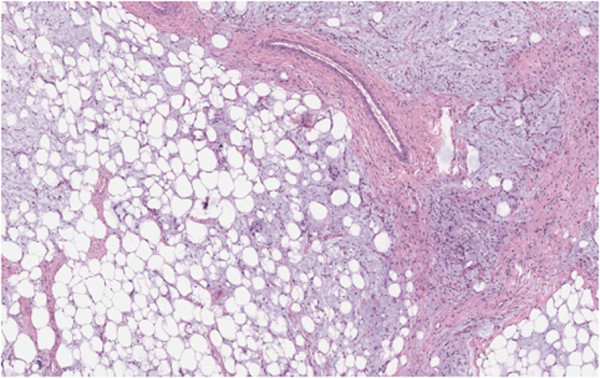
**Scattered among mature adipocytes, atypical spindled cells and a lipoblast are shown.** Note the variation in the size of the fat cells, a common feature in atypical lipomatous tumors (hematoxylin and eosin ×100).

**Figure 4:**
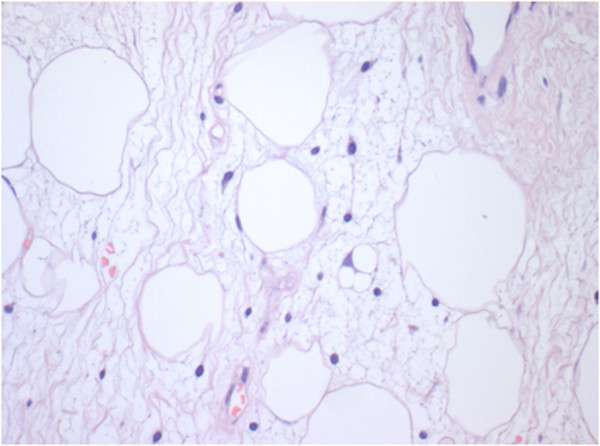
**Atypical spindle cells and a multivacuolated lipoblast are more evident at higher magnification (hematoxylin and eosin X400).**

Histologic findings showed the presence of liposarcoma of mixed type (myxoid liposarcoma with areas of well-differentiated ‘lipoma-like’ liposarcoma) according to World Health Organization (WHO) recommendations (Figures 3 and 4).

No postoperative chemotherapy or radiotherapy was administered.

At 24-month follow-up, the patient is doing well, with no clinical or radiological evidence of disease recurrence.

## Conclusions

PLs are rare tumors, 161 cases have been reported in the literature worldwide [6, 7] and typically reported as isolated cases or as a component of larger studies of liposarcomas [1, 2, 8–10]. Those affected were adult patients, aged 50 to– 60 years, with a range of 16 to –82 years [3] and involved the spermatic cord (76%), testicular tunics (20%) and epidydimis (1.4%).

Most PLs are slow-growing tumors, with a huge palpable scrotal or inguinal mass (sized between 1.5 and 20 to– 30 cm in diameter), occasionally accompanied by slight to moderate pain and a sensation of heaviness. Due to this clinical presentation, differential diagnosis should include scrotal lipoma, groin hernia, hydrocele and epididymitis [3, 7]. Rapid growth, larger size and pain suggest the presence of malignancy. In all cases of suspicion of malignancy, a physical examination should be followed by US and/or computed tomography (CT) scans or magnetic resonance imaging (MRI).

On US scans, PLs are identified as heterogeneous solid, hypoechoic lesions with central colliquation due to necrosis, in the case of larger tumors. In smaller ones, US scans cannot always distinguish PLs from benign lipomas, as in the case we present. CT scans show the lower density of PLs compared to subcutaneous fatty tissue [3, 4].

There is a correlation between tumor grade and histology and clinical behavior.

PLs are often classified in four subtypes:

- well-differentiated
- myxoid and round cell
- pleomorphic
- de-differentiated

Most of these tumors (about 40 to- 50%) are well-differentiated (lipoma-like), sclerosing and inflammatory, according to current criteria [2, 3, 7]. These tumors show slow growth and good prognosis, even in the case of larger lesions [2], after complete and extensive removal, while they tend to recur and metastasize when incompletely excised [5]. On the other hand, pleomorphic and dedifferentiated PLs, the rarest subtypes, are considered high-grade sarcomas, with a high rate of recurrence and metastasis, thus requiring a more extensive surgical and adjuvant treatment [11–14].

The term atypical lipomatous tumor embraces neoplasms with similar histological features but different clinical behavior, strongly influenced by location.

Subcutaneous and intramuscular/intermuscular tumors do not metastasize and the rate of recurrence and dedifferentiation is very low, particularly in the former. In these cases, they are designated ‘atypical lipoma’.

On the other hand, those located in central body sites are classified as ‘well-differentiated liposarcoma’ because they have high potential for recurrence and dedifferentiation. Although they can metastasize only when dedifferentiated, they can cause patient death simply by uncontrolled local growth.

The differential diagnosis from lipoma, spindle cell lipoma and dedifferentiated liposarcoma is based on light microscopy examination because immunohistochemistry is of little value in distinguishing among these neoplasms.

Implied in the foregoing statements, the most important role of the pathologist is to convey to the clinician the anticipated behavior of the lesion and the long-term risk of dedifferentiation.

Difficulty in preoperative diagnosis of PLs can affect the correct surgical treatment. A correct preoperative diagnosis is infrequent, due also to the very low incidence of these tumors. For this reason, a surgical approach can be incorrect, or even incomplete. In these cases, the incidence of local recurrence is very high. The recurrence of well-differentiated PLs often involves dedifferentiated PLs, with worsening prognosis.

Surgical treatment of PLs is radical orchiectomy and funicolectomy via the inguinal approach (with no need to perform local or retroperitoneal lymphoadenectomy, due to the lack of lymphatic spreading of these tumors), with wide excision of involved local tissues.

In our case, undoubtedly the tumor was a rare form of liposarcoma of mixed type ‘*ab initio*’. Relapse after a few months of a myxoid liposarcoma with areas of well-differentiated histotype cannot be explained otherwise. The histological misdiagnosis was made on the sample of the first surgical intervention. This may be attributed to one of the two followings factors: (1) sampling errors, myxoid areas were not identified and sampled for histological examination; (2) myxoid areas were a small part of the tumor and were not removed during the first surgical procedure.

To date, due to the rarity of PLs, the role of radiotherapy and chemotherapy has not yet been well established [3, 7]. The literature researches show controversial results achieved after radiotherapy and chemotherapy, mainly based on doxorubicin [1, 15]. This seems to be mainly due to the lack of consistent case series. For these reasons, wide surgical excision remains the only curative treatment. It should be proposed even in cases of smaller, well-differentiated tumors and also in younger patients, because of the high risk of local recurrence (and consequent tumor dedifferentiation) after inadequate surgery.

Paratesticular liposarcomas are rare tumors often located in paratesticular and spermatic cord tissues. When there is pre- or intraoperative diagnosis, radical orchiectomy and funicolectomy via the inguinal approach must be performed. In cases of incomplete surgery, we suggest performing an immediate radical procedure. The low response to adjuvant treatments imposes extensive surgery as the only curative approach, even in cases of smaller and well-differentiated tumors, due to the high risk of local recurrence in cases of nonradical excision.

## Consent

Written informed consent was obtained from the patient for publication of this Case report and any accompanying images. A copy of the written consent is available for review by the Editor-in-Chief of this journal.

## Abbreviations

CT: computed tomography
PL(s): paratesticular liposarcoma(s)
MRI: magnetic resonance imaging
US: ultrasound.
